# Assessment of Photodynamic Therapy Penetration Depth in a Synthetic Pig Brain Model: A Novel Approach to Simulate the Reach of PDT-Mediated Effects In Vitro

**DOI:** 10.3390/ph18121837

**Published:** 2025-12-02

**Authors:** Nicolas Bader, Annika Hajosch, Christian Peschmann, Kathrin Stucke-Straub, Christian Rainer Wirtz, Richard Eric Kast, Marc-Eric Halatsch, Felix Capanni, Georg Karpel-Massler

**Affiliations:** 1 Biomechatronics Research Group, Ulm University of Applied Sciences, 89075 Ulm, Germany; felix.capanni@thu.de; 2 Department of Neurosurgery, Ulm University Medical Center, 89081 Ulm, Germany; annika.hajosch@uniklinik-ulm.de (A.H.); rainer.wirtz@uniklinik-ulm.de (C.R.W.); georg.karpel-massler@uniklinik-ulm.de (G.K.-M.); 3 Department of Anesthesiology and Intensive Care, Ulm University Medical Center, 89081 Ulm, Germany; christianpeschmann@gmail.com; 4 Department of Mathematics, Natural and Economic Sciences, Ulm University of Applied Sciences, 89075 Ulm, Germany; kathrin.stucke-straub@thu.de; 5 IIAIGC Study Center, Burlington, VT 05408, USA; richarderickast@gmail.com; 6 Department of Neurosurgery, Cantonal Hospital of Winterthur, 8401 Winterthur, Switzerland; marc-eric.halatsch@ksw.ch; 7 Advanced Treatment Concepts Against Glioblastoma (ATCG), 8280 Kreuzlingen, Switzerland

**Keywords:** photodynamic therapy, glioblastoma, synthetic pig brain optical substitute, therapeutic light penetration depths, 5-ALA

## Abstract

**Background/Objectives:** Recurrence of glioblastoma (GBM) mostly occurs in close vicinity to the resection cavity. Therefore, our group has previously designed an implant to locally apply repetitive photodynamic therapy to mitigate tumor recurrence. The penetration depths of different wavelengths in brain tissue were exhaustively studied before. However, the PDT-induced biological effects of 5-ALA-based PDT against GBM cells at different depths have not been evaluated yet. **Methods:** Therefore, a synthetic brain substitute material of 1–10 mm thickness and with optical properties comparable to the white or gray matter of pig brain was developed. Tumor cell viability was assessed in spheroids from six GBM cell lines using disks of varying thickness prepared from pig brain substitute material to mimic in vivo radiation attenuation. **Results:** Using an artificial brain tissue optical model based on material science, we have established a relationship between the PDT-induced effect of our PDT implant and the distance of migrating GBM cells from the resection cavity wall. **Conclusions:** This model may be helpful to aid optimization of the irradiation doses and fractionation required to attain the maximal therapeutic effect by long-term PDT applications.

## 1. Introduction

Glioblastoma (GBM) has an incidence of approximately 3.22 per 10,000 inhabitants in the United States and represents the most common primary brain tumor in adults [1]. Due to its infiltrative behavior and its incalcitrant nature, GBM can be considered a basically incurable disease. This notion is reflected by a median overall survival (OS) of usually less than 2 years after diagnosis despite safe maximal resection, radio- and chemotherapy with temozolomide as well as alternating electric fields [2]. 5-Aminolevulinic acid (5-ALA)-based photodynamic therapy (PDT) has emerged as an adjuvant therapeutic measure and is currently being investigated in clinical trials [1,3,4,5,6,7,8,9]. 5-ALA is a precursor in the heme biosynthesis pathway. Once inside the cells, 5-ALA is metabolized into the photosensitive molecule protoporphyrin IX (PpIX), which selectively accumulates in malignant cells such as GBM. PpIX has the property of capturing photon energy in form of light of specific wavelengths with the highest absorbances at 405 and 630 nm [10,11]. The release of this energy leads to fluorescence or to the generation of reactive oxygen species (ROS) in tumor cells. The most common ROS produced by PDT are singlet oxygen (^1^O_2_) and superoxide anion ($O_{2}^{•-}$) [12,13]. Oxidative stress induced by ROS triggers apoptosis through two primary pathways. The first is the extrinsic pathway, where the Fas receptor is activated and binds with FADD and procaspase-8 to form a death-inducing signaling complex (DISC) [14,15]. This complex cleaves procaspase-8 into caspase-8, which in turn activates caspase-3 and caspase-7 [16]. Caspase-3 facilitates DNA fragmentation by releasing caspase-activated deoxyribonuclease (CAD) from its inhibitor ICAD, while caspase-7 contributes to cellular protein degradation [17]. The second is the intrinsic pathway, where the activity of the photosensitizer in PDT leads to the inhibition of Bcl-2 and an increase in BAX protein expression. This causes increased mitochondrial outer membrane permeability, leading to the release of cytochrome c into the cytoplasm. Cytochrome c then binds with APAF1 and procaspase-9, forming apoptosomes that cleave caspase-9. This initiates further activation of caspase-3, resulting in apoptosis through DNA fragmentation and protein degradation [18,19,20,21]. The very short lifetime of singlet oxygen of less than 320 ns allows just a small diffusion range of the molecule of only up to 55 nm within the cells, rendering PDT a tumor-selective treatment without damaging sensitive healthy tissues [13,22]. More recent measurements, however, reported lifetimes of 15–30 μs, with corresponding diffusion radii of approximately 155 nm in neat water and up to 550 nm in deuterated water [23]. Considering that the typical eukaryotic cell diameter is about 10–30 μm, singlet oxygen can still be regarded as acting locally at the subcellular level, largely confined to organelles, rather than diffusing across entire cells. Superoxide anion generation in itself can also be enhanced by photosensitizers like 5-ALA, increasing oxidative phosphorylation without conversion to PpIX [13,23].

While PDT holds potential as a therapeutic adjunct for the treatment of brain tumors, it faces several limitations. Among its most important constraints are depth restriction and the confinement of treatment to the intra-operative setting during surgery. To overcome these limitations, our group has previously developed a first prototype of an implant for a tolerability study using light-emitting diodes (LEDs) with a peak wavelength of 630 nm, allowing for longer treatment duration and enabling highly flexible and diverse irradiation regimens [24]. Most approaches using PDT for the treatment of GBM rely on laser technology because of its comparatively high energy output. However, due to their small size, LEDs are more feasible for a long-term intracerebral implant despite their lower energy output. Coherency of the light source is neither a requirement for successful PDT nor does it affect the efficiency of PDT [12,24,25]. In a first safety study, we have tested our PDT implant for tolerability in a porcine model. PDT applied with a fluence rate of 19.5 mW/cm^2^ for 30 min daily over 14 d or continuously for 14.6 h was shown to be well tolerated by the animals [26].

PDT relies on the tissue penetration of light into tissue which is shallow and typically reaches only a few millimeters. The purpose of this study was to generate and validate an in vitro model simulating the optical properties of porcine brain to evaluate the therapeutic response of our PDT implant depending on the depth of light penetration through brain tissue. In this work, the term PDT-induced biological response refers to the depth-dependent cellular effects resulting from photodynamic therapy within our in vitro experimental model, which combines an optical brain phantom with three-dimensional tumor spheroids. This term does not imply clinical efficacy or tumor eradication. To the best of our knowledge, this is the first model of this kind [19,20,21].

## 2. Results

### 2.1. The Phantom Materials Attenuate Both Irradiance and the Inhibitory Effects on Spheroid Growth in a Thickness-Dependent Manner

To assess how the phantoms emulating the optical characteristics of porcine brain affect the passing of light through the absorbing material, irradiance measurements were conducted. For this purpose, a light source emitting at 42.25 mW/cm^2^ and 630 nm wavelength was positioned behind phantom plates mimicking white matter with thicknesses of 1, 2, 3, 4, 5, and 6 mm, as well as gray matter with thicknesses of 1, 2, 3, 5, 7, and 9 mm. These measurements showed an exponential decline of irradiance depending on the thickness of the substitute material that was passed by the light. The data are presented in Table 1.

**Figure 1:**
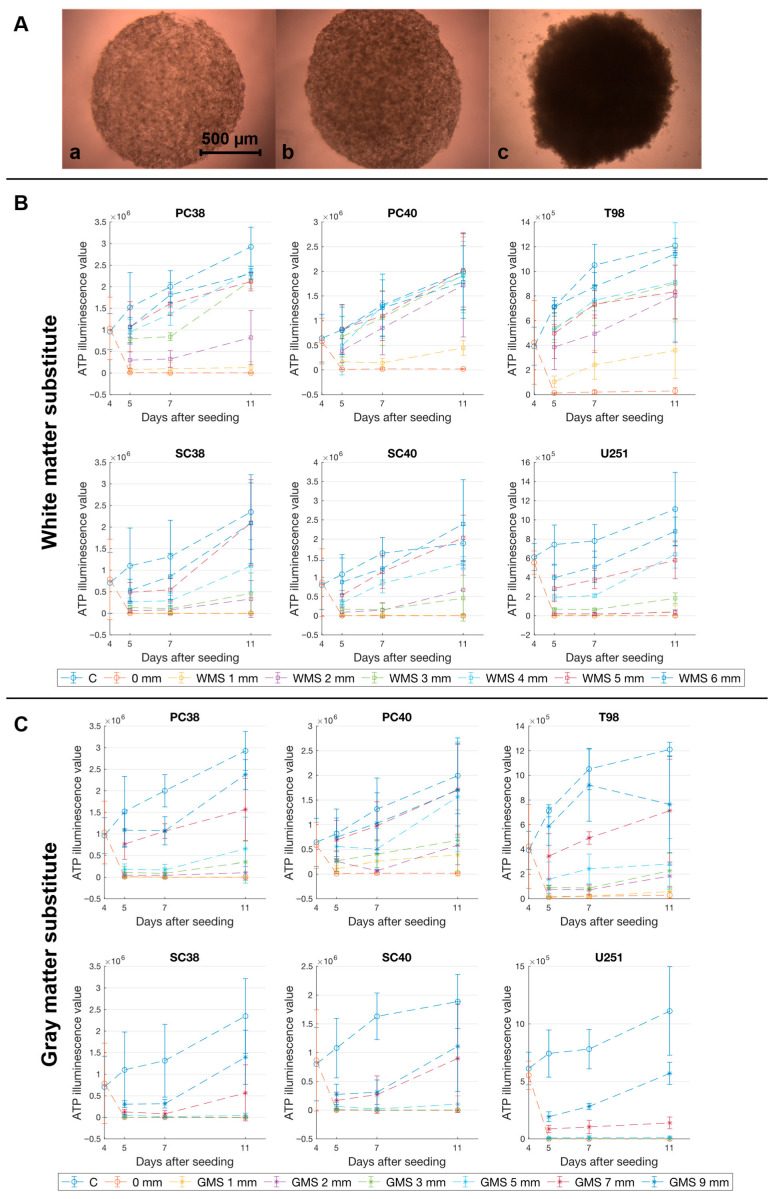
(**A**) Representative microphotographs of spheroids derived from PC40 cells on d 5 (**a**), d 7 (**b**), and d 11 (**c**), showing the progressive proliferation of the spheroids over time. Magnification 4 ×. (**B**,**C**), Spheroids were allowed to form for 4 d prior to irradiation. ATP illuminescence, serving as an indicator of cell viability was determined on d 5, 7 and 11. Data are presented as mean with standard deviation from three independent experiments. (**B**) shows the results for white matter substitute (WMS), and (**C**) for gray matter substitute (GMS). Both panels illustrate the course of ATP luminescence in all GBM cell lines used, starting from day 4 for untreated controls (**C**) and the direct irradiation group (0 mm), or from day 5 for all groups with phantom plates of different thicknesses as specified in the respective legends. The inhibitory effect of PDT can be recognized in several treatment groups, whereas proliferation is evident in the controls and in groups with thicker phantom plates.

We generated our model as a means to assess the potential biological effects of PDT dependent on the distance that light has to travel through brain tissue before it reaches tumor cells. For that purpose, spheroids with a diameter of approximately 1.5 mm were used, allowing cellular proliferation within, prior to exposure to PDT attenuated by the penetration of light through optical phantoms with increasing thickness. Growth curves for spheroids treated with control (solvent) were determined and used to ensure application of PDT during the spheroids’ exponential growth phase (Figure 1).

Cell viability is intricately tied to the intracellular ATP levels within each spheroid [27,28]. Figure 1B,C illustrate the development of mean ATP illumination values in 3-dimensional GBM in vitro models in response to PDT. Increasing thickness of the optical phantom material resulted in a reduction in the inhibitory effect on cell viability across all the GBM spheroids tested (Figure 1B,C). Notably, the inhibition of spheroid viability was less pronounced for brain substitute material mimicking the optical properties of gray matter when compared to white matter substitute of equal thickness.

Spheroids derived from PC38 cells showed a PDT-induced biological response when irradiated through gray matter substitute up to a distance of 9 mm, whereas white matter substitute permitted detectable PDT-induced effects only up to a thickness of 5 mm. Similar patterns were observed for all GBM spheroid models tested.

### 2.2. PDT Predominantly Exerts a Sustained Inhibitory Effect on Spheroid Growth over Several Days

To further assess the biological effect of PDT on the growth of spheroids over time and in dependence of the thickness of the optical phantoms, we defined three distinct zones of PDT-induced biological response.

**Figure 2:**
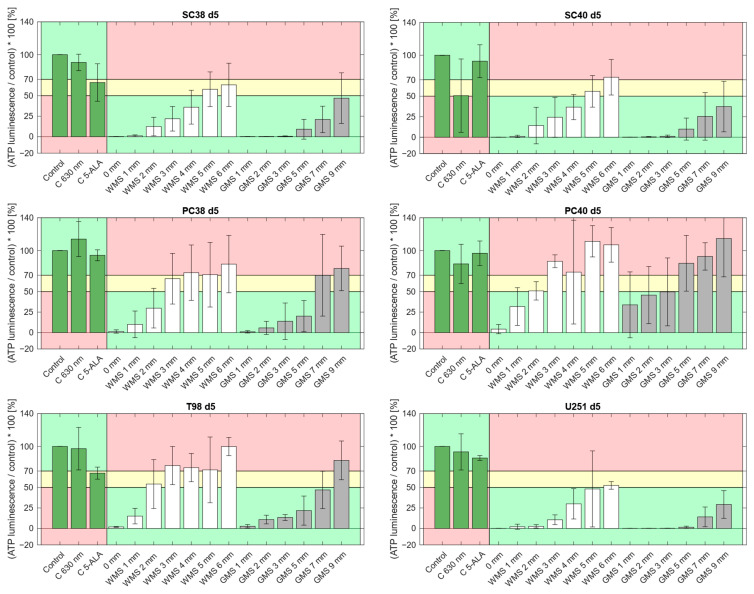
Spheroids of the indicated GBM cells were allowed to form for 4 d prior to irradiation with 630 nm and 42.25 mW/cm^2^ through optical phantoms of the indicated thickness. PDT-induced biological responses 1 d after irradiation are shown. Data are normalized to control (C) and presented as mean ± standard deviation from three independent experiments. Control conditions were: C = untreated spheroids; C 630 nm = irradiation in the absence of 5-ALA; C 5-ALA = 5-ALA in the absence of irradiation. Optical phantom conditions included white matter substitute (WMS) and gray matter substitute (GMS). The values in mm indicate the thickness of the respective phantom plates, while “0 mm” refers to direct irradiation in the presence of 5-ALA without any optical phantom. The No Effect zone is defined by an ATP luminescence above 70% (indicated in red); the Consider zone is defined by a range from 50% to 70% (indicated in yellow), and the PDT Effect zone is defined by an ATP luminescence below 50% (indicated in green). For the three control groups, the color coding was switched between the PDT Effect and the No Effect zone.

For white matter substitute material, the thickness threshold for a PDT-induced biological response to PDT 1 d after irradiation (d 5 after spheroid seeding) was 4 mm for SC38 as well as SC40 and 5 mm for U251 cells (Figure 2). Adding the depths of the respective Consider zones to those distances resulted in an expanded thickness threshold of 6 mm for a PDT response. For PC38, PC40 and T98G cells, penetration depth thresholds for a PDT response were lower.

**Figure 3:**
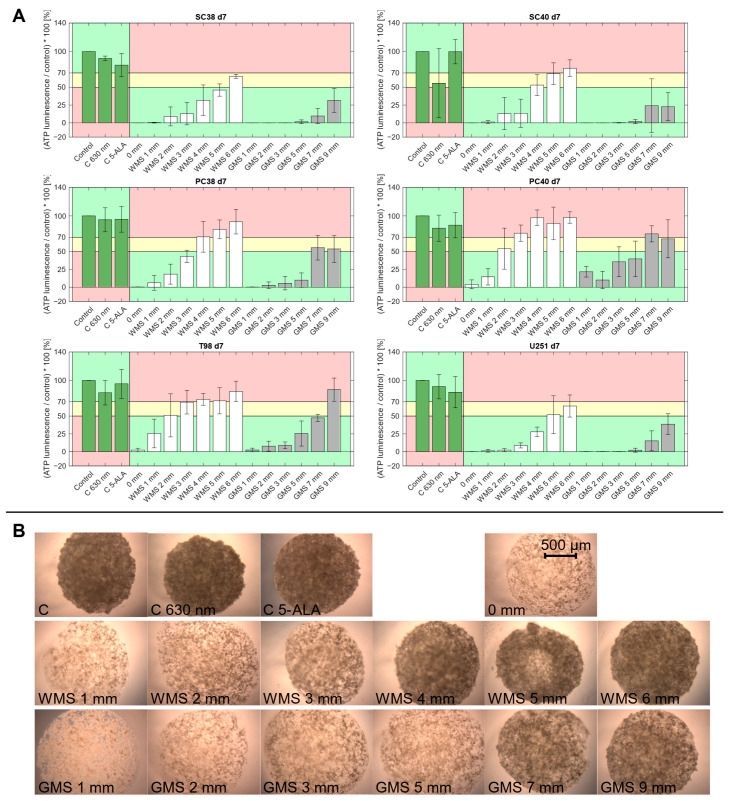
(**A**) Spheroids of the indicated GBM cells were allowed to form for 4 d prior to irradiation with 630 nm and 42.25 mW/cm^2^ through optical phantoms of increasing thickness. PDT-induced biological responses 3 d after irradition are shown. Data are normalized to untreated controls (C) and presented as mean ± standard deviation from three independent experiments. Control conditions were: C = untreated spheroids; C 630 nm = irradiation in the absence of 5-ALA; C 5-ALA = 5-ALA in the absence of irradiation. Optical phantom conditions included white matter substitute (WMS) and gray matter substitute (GMS). The values in mm indicate the thickness of the respective phantom plates, while “0 mm” refers to direct irradiation in the presence of 5-ALA without any optical phantom. The No Effect zone is defined by an ATP luminescence above 70% (indicated in red); the Consider zone is defined by a range from 50% to 70% (indicated in yellow), and the PDT Effect zone is defined by an ATP luminescence below 50% (indicated in green). For the three control groups, color coding was switched between the PDT Effect and the No Effect zones. (**B**), Representative microphotographs of spheroids derived from PC38 cells on d 7 (3 d post PDT). The indicated conditions correspond to those shown in panel A. Proliferation, and thus the PDT-induced biological response, can be directly recognized in these microphotographs that are consistent with the ATP-based viability measurements. Magnification 4×.

Notably, in gray matter substitute material we observed greater PDT penetration depths compared to white matter phantoms. On d 5, three out of the six GBM models we examined (SC38, SC40 and U251) maintained PDT-induced biological responses within the tested range of up to 9 mm. PDT effects up to a depth of 7 mm were seen in T98G cells, the No Effect zone was reached only at a thickness of 9 mm. A slightly lower PDT effect was observed in PC38 cells showing a biological response up to a depth of 5 mm, passing over to the Consider zone at 7 mm and advancing into the No Effect zone at 9 mm thickness. The least PDT effect was found in PC40 cells with that effect being observed only up to 3 mm. Similar results were obtained on d 7 (Figure 2 and Figure 3).

**Figure 4:**
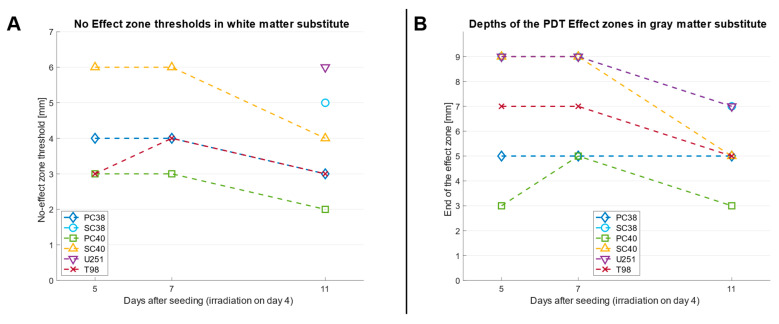
(**A**), Shifts in the No Effect zone in GBM spheroids over time with white matter substitute. This zone is defined as the minimum phantom material depth beyond which no PDT-induced biological response is observed. The analysis also includes the adjacent Consider zone, representing partial PDT-induced biological responses with ATP luminescence values from 50 to 70 %. Measurements were taken 1, 3, and 7 d after PDT (630 nm, 42.25 mW/cm^2^) applied on d 4 after spheroid seeding through tissue phantoms mimicking the optical properties of white matter. (**B**), Shifts in the PDT Effect zones in the same experimental setup using gray matter substitutes. Here, the maximal depth allowing a PDT response was assessed by tracking the deepest point at which a measurable biological effect was still observed, marking the transition to the Consider zone. Data represent mean values from three independent experiments.

As shown in Figure 4A, the depth at which no PDT-induced biological response was detectable, referred to as the No Effect zone, remains largely unchanged for most cell models between d 5 and 7. However, between d 7 and d 11, a post hoc decrease in the PDT penetration depth was noted in that the onset of the No Effect zone shifted towards the light source, indicating that single treatments became less effective at deeper tissue levels over time. In PC38 and T98G cells, the start of this zone moved from 4 mm to 3 mm, and in PC40 cells from 3 mm to 2 mm. For SC40 cells, the shift was even more pronounced, from 6 mm to 4 mm (Figure 4A). In contrast, SC38 and U251 cells showed full PDT-induced biological responses up to 6 mm until d 7. A No Effect zone became apparent only as of d 11, starting at 6 mm in U251 and at 5 mm in SC38. Since white matter phantoms were only tested up to a thickness of 6 mm, the full extent of the gradual loss of PDT effectiveness in this context could not be determined with the current experimental setup.

For gray matter substitute, the overall PDT effect was considerably stronger. As a result, only few experimental treatments fell into the Consider or No Effect zones. Therefore, instead of tracking the onset of the No Effect zone as performed for white matter substitute material, the shifts in the PDT Effect zones were analyzed by tracking the deepest point at which a clear PDT-induced biological response was observed, corresponding to the transition to the Consider zone (Figure 4B). Between d 5 and 7, the PDT penetration depth remained largely stable across most GBM models. An exception was PC40 cells which showed an increase in the PDT Effect zone depth from 3 mm to 5 mm, indicating delayed response.

Again, by d 11, reduced t PDT-induced biological effects became evident in nearly all models. U251 cells showed a decrease in PDT Effect zone depth from 9 mm to 7 mm, T98G cells from 7 mm to 5 mm, and PC40 cells from 5 mm to 3 mm. A more pronounced decline was observed in SC40 cells, with the PDT Effect zone depth decreasing from 9 mm to 5 mm. In contrast, PC38 cells maintained a stable PDT Effect zone depth of 5 mm across all measured time points. In the case of SC38 cells, the PDT effect initially exceeded the upper detection limit of 9 mm on d 5 and d 7, but was reduced to a thickness of 7 mm on d 11.

## 3. Discussion

While 5-ALA is widely accepted and used for fluorescence-guided resection of GBM, its potential as a therapeutic adjunct in the context of 5-ALA-based PDT of malignant brain tumors has only started to be explored. Clinical trials have shown potential merit of PDT in this setting. The INDYGO trial, a prospective, non-randomized, single-center phase I study, applied a standardized therapeutic approach consisting of 5-ALA HCl fluorescence-guided surgery followed by intraoperative PDT using a single 200 J/cm^2^ light dose, with patients subsequently receiving adjuvant therapy according to the Stupp protocol. The study demonstrated a median OS of 23.4 months as well as 2-year and 5-year OS rates of 50% and 40%, respectively, for patients with newly diagnosed GBM. Median progression-free survival (PFS) was 17.1 months with a 12-month PFS rate of 60%. At the latest follow-up (November 2023), four out of 10 patients were still alive, including one without recurrence. Importantly, no severe adverse effects or treatment-related deaths occurred, and quality of life metrics remained stable or improved in most patients [29,30]. Currently, a multicenter phase II clinical trial is recruiting patients with recurrent GBM to evaluate the safety and efficacy of stereotactic PDT in a randomized controlled fashion (NOA11, NCT04469699). This study is expected to be completed in 2026.

PDT faces several limitations. Especially when applied to brain tumors, two major problems are confronted: (1) The intraoperative duration of the treatment is restricted, and (2) Light passing through brain tissue is attenuated before it reaches the tumor cells, and is further attenuated while passing through tumor tissue itself. In an attempt to address these limitations, our group has developed an implantable device capable of delivering light of specific wavelengths to the tumor cell-infiltrated brain from within an intracerebral tumor resection cavity, termed Globus Lucidus [26]. To examine how this implant may affect the viability of tumor cells depending on the penetration depth of light in brain tissue, we generated the synthetic model presented here. Overall, the experimental setup described in this work uses relevant technical parameters that ultimately correspond to the aforementioned implant (albeit in a different configuration), thus allowing indirect validation of the implant’s radiation parameters against GBM spheroids.

Our measurements have shown that the optical properties of the phantom material created for this study are equal to those of white and gray matter of porcine brain for 630 nm light, the relevant wavelength for PDT. The brain tissue substitute was also designed to emulate the optical properties for 405 nm, which is another absorption peak of PPIX, and thus can be used for studies with this wavelength as well. Although the synthetic phantom reproduces the optical properties of brain tissue, it does not reflect biological or systemic characteristics such as cellular responses, vascularization, perfusion, immune interactions, or hypoxia and therefore cannot model the interaction between PDT effects and the surrounding biological environment. It remains useful primarily for evaluating light propagation and selecting PDT parameters. The substitute material can be easily machined on a lathe and adjusted to the required size. The storage and handling of the material as well as the cleaning of the experimental setup were less complex compared to the use of original brain tissue. As expected, the irradiance behind a 1 mm thick phantom material layer is already markedly reduced, confirming the low penetration depth of light with 630 nm wavelength within the brain. Gray matter allows a slightly better penetration of light of this wavelength relative to white matter. When comparing the optical properties of our brain tissue substitute material with the data from Mosca et al. [31], a stronger agreement was observed for the absorption coefficient μ_a_ than for the reduced scattering coefficient μ_s_′. Mosca et al. conducted their measurements in the 650–1100 nm range; for results within these confines, refer to the work of Bergmann et al. The different results regarding μ_s_′ values in Mosca et al.’s data versus our findings are likely due to differences in sample composition. While our samples were each separated into two individual components, Mosca et al. measured the optical properties of the whole brain tissue, i.e., both gray and white matter combined. A similar pattern was noted when comparing the scattering coefficient with the work from Jacques et al. [32]. In contrast, Schwarzmaier et al. [33] measured the optical properties of white and gray matter separately, yielding results comparable to ours except for the μ_a_ values for white matter which were slightly higher in their study than in ours.

We chose to assess the biological efficacy of PDT in a three-dimensional setting using spheroids in order to more accurately simulate and reflect the real-world scenario compared to a two-dimensional single-layer cell culture model. Along this line, the cell cultures used were derived from patients operated on in our institution. Moreover, intratumoral heterogeneity as a common feature of GBM was addressed by using spheroids generated from multiple genetically diverse cultured GBM cells, including cells with stem-like features. Despite these efforts to more closely align our model with real-world conditions, it encounters limitations and undoubtedly captures only a fraction of the complexity of the clinical situation. Furthermore, ATP-based luminescence measurements were used as a surrogate for cellular viability, it should be noted that this method detects both cytotoxic and cytostatic states and therefore does not directly discriminate between different modes of cell death. As outlined by Forgie et al. [34], ATP-based viability assays reflect the functional and energetic status of cells rather than survival in the strict clonogenic sense. However, the cellular mechanisms induced by photodynamic therapy are well characterized and predominantly cytotoxic, driven by the formation of reactive oxygen species that trigger apoptosis and necrosis. In this context, a reduction in ATP content is therefore most likely attributable to cytotoxic effects. The depth-dependent viability changes observed in this study are consistent with necrosis depths reported in clinical PDT literature and support the suitability of this approach for establishing an in vitro model that incorporates tissue-like optical attenuation [35]. It must be acknowledged that ATP measurements cannot assess long-term survival or eradication, and future investigations involving extended irradiation protocols or deeper penetration depths should include complementary methods such as clonogenic assays to confirm long-term treatment effects. Nevertheless, the model may serve as a useful tool for improving predictions of therapeutic response and guiding further exploration to identify measures for refining treatment conditions.

Importantly, our in vitro approach also aligns with internationally accepted 3R principles (Replacement, Reduction and Refinement). While the in vivo reach of PDT-mediated effects is ultimately the clinically relevant parameter, establishing a controlled and reproducible in vitro basis allows systematic evaluation of key irradiation parameters such as fluence rate, exposure duration, repetition frequency and timing intervals before initiating animal studies. This strategy helps reduce the number of required in vivo experiments and ensures that only the most promising and biologically meaningful parameter sets are advanced to subsequent preclinical testing, thereby minimizing unnecessary animal use.

In this study, our data show that the PDT penetration depth of a single PDT session with light of 630 nm and an irradiance of 42.25 mW/cm^2^ over 60 min, yielding a radiant exposure of 152.1 J/cm^2^, varies among the cell lines used here. With gray matter substitute and 1 d after irradiation, the majority of the spheroid models showed a PDT-induced biological response beyond a light penetration depth of 5 mm and up to more than 9 mm. This radiant exposure would be sufficient to overcome the layer of human gray brain matter which on average measures between 2 and 4 mm [36], potentially allowing both tolerable and effective PDT of tumor-infiltrated cortex from a subcortical resection cavity.

Our results for white matter substitute split into two groups. The stem-like cells SC38 and SC40 (as well as U251) cells responded to PDT in greater light penetration depths of 4–5 mm compared to the primary cultures PC38 and PC40 (as well as T98G) cells which showed relevant biological effects in maximal penetration depths of only 1–3 mm. These data suggest that cells with stem-like features may be more responsive to PDT and stress the potential benefit of integrating this treatment into a multimodal approach to specifically target the stem cell niche. Overall, the PDT penetration depths that were observed in our work are consistent with findings from several clinical studies as summarized by Quirk et al. who also reported PDT-induced necrosis to predominantly occur at depths of up to 7 mm around the resection cavity wall [35].

Clinical studies report that in most patients, recurrence occurs within 1–2 cm of the resection cavity wall within the first year after initial treatment [12,37]. Therefore, when we conceptualized an implant for adjuvant GBM treatment, we aimed at therapeutic penetration depths reaching or exceeding 20 mm. Our data show that penetration depths of this extent are unlikely to be reached by a typical single session of PDT. However, an implant allows for repetitive irradiation which could cumulatively increase the therapeutic penetration depth by successively applying more radiant exposure to the targeted tissue. Further studies are warranted to define the optimal conditions to reach the highest possible therapeutic penetration depth. Based on data from other groups [38,39,40,41], such refinement of the treatment conditions may indeed result in improved PDT-induced biological response. For example, Mathews et al. demonstrated in vitro that continuous PDT of only 17 µW/cm^2^ over 24 h was sufficient to induce significant effects [39]. These findings underline the potential of low-dose, prolonged irradiation strategies and highlight the importance of further investigating implant-based delivery systems in future studies, as such systems could effectively implement these long-term irradiation regimens.

## 4. Materials and Methods

### 4.1. Cell Cultures and Growth Conditions

U251MG and T98G human GBM cell lines, sourced from the American Type Culture Collection (Manassas, VA, USA), were cultured in Dulbecco’s modified Eagle’s medium (DMEM; GIBCO, Invitrogen, Paisley, UK) supplemented with 10% fetal bovine serum (FBS), 100 IU/mL penicillin, 100 µg/mL streptomycin, 4 mM glutamine, and 1 mM sodium pyruvate (GIBCO, Invitrogen, Grand Island, NY, USA) as previously detailed [42]. Initial stocks of these cell lines were expanded, frozen, and stored in liquid nitrogen, with fresh aliquots thawed every 6 weeks.

SC38 and SC40 are primary cultured human glioma stem-like cells derived from tumor resections carried out at the Ulm University Medical Center as previously described [43,44]. The glioma stem-like phenotype of SC38 and SC40 cells was preserved by maintaining cells as sphere cultures in DMEM/F-12 (HAM) medium (Gibco, Life Technologies, Darmstadt, Germany) enriched with serum-free neuron culture supplement B27 (Gibco, Life Technologies), human recombinant epidermal growth factor (Biomol GmbH, Hamburg, Germany) and human recombinant basic fibroblast growth factor (Miltenyi Biotec GmbH, Bergisch Gladbach, Germany). Differentiated cells (PC38 and PC40) were derived from the respective glioma stem-like cell spheres by allowing adherence and growth in DMEM in the presence of 10% FBS. PC38 and PC40 were maintained for a maximal duration of 10 weeks. All cell lines were incubated at 37 °C in a water-saturated atmosphere containing 5% CO_2_. An overview of all glioblastoma cell models, including their origin, patient characteristics, and molecular properties, is provided in Table 2.

The procedures were approved by the ethics committee of the University of Ulm (No. 162/10) and consent was granted by the patients or next of kin.

### 4.2. Spheroid Assay

To evaluate the effects of PDT in three-dimensional growth settings, spheroids with a diameter of 1.5 mm were produced in batches of 20 with 1.5 × 10^5^ cells for U251, T98G, PC38 as well as PC40 and 3 × 10^4^ cells for SC38 and SC40, then resuspended in 200 µL of a mixture of 80% Matrigel and 20% DMEM. Subsequently, 20 aliquots of 10 µL each from this suspension were deposited into V-bottom 96-well plates, containing 7.5 × 10^3^ cells of U251, T98G, PC38 and PC40 and 1.5 × 10^3^ cells of SC38 and SC40.

After 30 min at room temperature, the cell/Matrigel matrix was gently transferred into 12-well plates containing the respective culture medium, i.e., DMEM supplemented with 10% FBS for U251, T98G, PC38, and PC40, and stem cell medium (DMEM/F-12 supplemented with B27 and growth factors) for SC38 and SC40. Spheroids were allowed to grow for a period of 4 d. Following treatment on d 4, the spheroids were maintained in the corresponding culture medium containing reduced serum supplementation, i.e., DMEM with 1.5% FBS for U251, T98G, PC38, and PC40, or stem cell medium with B27 supplement for SC38 and SC40, with an additional medium change on d 7.

Quantification was performed by CellTiter-Glo^®^ assays on d 5, d 7 and d 11. To this purpose, spheroids were transferred to opaque-walled 96-well plates prior to adding 100 µL of medium and 100 µL of the CellTiter-Glo^®^ solution followed by 2 min of automated shaking and 10 min of incubation at room temperature. Then, measurement of luminescence was performed with a TriStar^2^ LB 942 Multimode Microplate Reader (Berthold Technologies GmbH & Co. KG, Bad Wildbach, Germany). Intracellular adenosine triphosphate (ATP) levels served as a surrogate for viable, metabolically active cells, since loss of membrane integrity and metabolic activity rapidly depletes cellular ATP pools [27,28]. In addition to ATP-based viability measurements, spheroids were inspected microscopically prior to each CellTiter-Glo^®^ assay. This enabled documentation of spheroid morphology and proliferative activity, providing complementary qualitative information on treatment-induced effects.

### 4.3. Optical Phantom Plates

**Figure 5:**
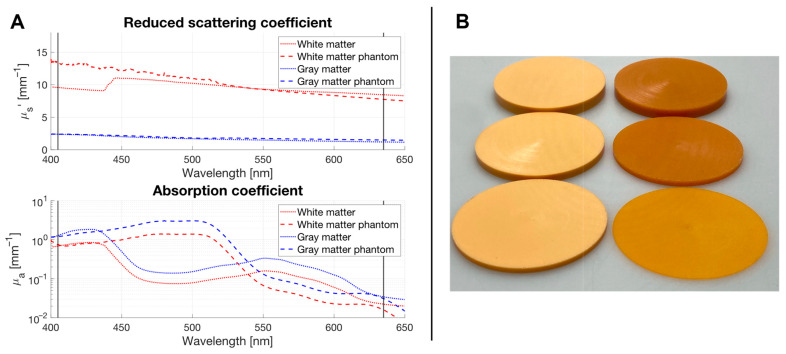
(**A**) Graphical representation of the reduced scattering and absorption coefficients for white and gray pig brain matter in comparison to the phantom materials. Notably, the phantom materials were specifically engineered to emulate the optical properties of pig brain matter at 405 and 630 nm. (**B**) Photographs of optical phantom disks of diverse thicknesses to represent both white (left) and gray (right) brain matter, each possessing optical properties for light at 405 nm and 630 nm that correspond to the respective brain tissue.

Optical property measurements on white and gray matter from pig brains were conducted in collaboration with the Institute for Laser Technologies in Medicine and Metrology at the University of Ulm as previously described by Bergmann et al. [46]. Based on these measurements, artificial materials were generated with matching optical properties at 405 nm and 630 nm for both white and gray pig brain matter. Figure 5A illustrates the reduced scattering coefficients μ_s_’ and absorption coefficients µ_a_ for white and gray pig brain matter as well as for the corresponding phantom material. The reduced scattering coefficient accounts for both the scattering coefficient (μ_s_) and the anisotropy factor (g) of tissue, and is defined as μ_s_’ = μ_s_ × (1 − g), describing the effective scattering that contributes to light diffusion in tissue. The respective values for white matter are (405 nm: μ_s_′ = 13.30 mm^−1^, μ_a_ = 0.75 mm^−1^; 630 nm: μ_s_′ = 7.82 mm^−1^, μ_a_ = 0.02 mm^−1^) and for gray matter (405 nm: μ_s_′ = 2.40 mm^−1^, μ_a_ = 1.21 mm^−1^; 630 nm: μ_s_′ = 1.51 mm^−1^, μ_a_ = 0.04 mm^−1^).

The phantom material was sectioned into plates with varying and precisely defined thicknesses. The plates were manufactured using a lathe, allowing a thickness precision of ±10 µm. Representative examples of these plates are shown in Figure 5B.

### 4.4. Photodynamic Therapy

**Figure 6:**
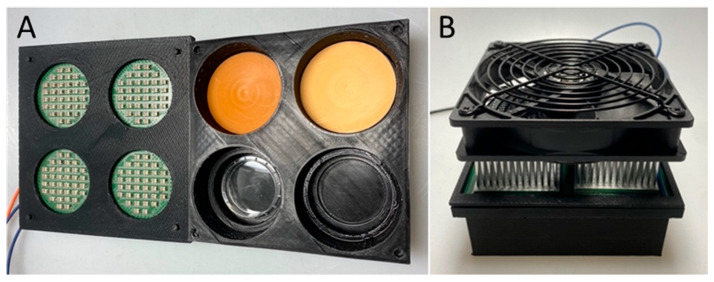
(**A**) Photograph showing the internal view on the radiation device for the delivery of PDT (with a wavelength of 630 nm and up to 42.25 mW/cm^2^ per chamber) which can accomodate optical phantom plates of various thicknesses between the light source and Petri dishes containing GBM spheroids. (**B**) Photograph showing the external view on the radiation device with the active cooling system to prevent overheating of the irradiation chambers.

A radiation device containing four irradiation chambers was designed to fit the optical phantom disks (Figure 6A,B). Each chamber contains 36 LEDs (150141RS63140, Würth Elektronik, Waldenburg, Germany) with a peak wavelength of 630 nm (dominant wavelength 620 nm, spectral bandwidth 20 nm) and a total irradiance of up to 42.25 mW/cm^2^. An active cooling system consisting of a heat sink and a fan was used to prevent overheating of the chambers (Figure 6B).

For PDT, spheroids were pre-treated with 5-ALA at a concentration of 100 µg/mL 4 h prior to light exposure. Irradiation was performed with the spheroids being resuspended in PBS for a total duration of 60 min, leading to a radiant exposure of 152.1 J/cm^2^. Optical phantom plates of varying thickness were positioned between the light source and the spheroids. Three control conditions were implemented in each experimental series: untreated spheroids (dark control), irradiation in the absence of 5-ALA (light control), and 5-ALA treatment in the absence of irradiation (ALA control). After irradiation, the spheroids were transferred into 12-well plates containing DMEM supplemented with 1.5% FBS.

### 4.5. Light Intensiy Measurements

All light intensity measurements were performed using the optical power monitor OPM 150 from QIOPTIQ^®^ (Qioptiq Photonics GmbH & Co. KG, Göttingen, Germany) and the OPM150 software (V. 1.15).

### 4.6. Statistical Analysis

**Figure 7:**
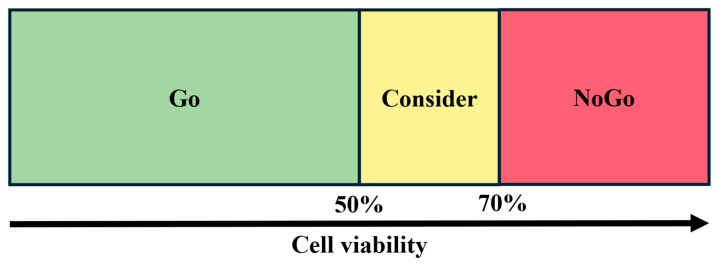
Visual representation of the Go/NoGo classification scheme divided into three zones. In this scheme, a reduction in ATP based viability reflects PDT-induced biological response. The PDT Effect zone (Go), defined by a viability of less than 50%, indicates a clear PDT effect. The No Effect zone (NoGo), defined by a viability of more than 70%, indicates insufficient response and thus no relevant PDT effect. The Consider zone, covering 50–70% viability, represents ambiguous outcomes, which were evaluated with additional qualitative information such as microscopic imaging and morphological observations. Cell viability data were normalized relative to the control group, allowing direct comparisons of the GBM cell spheroids’ PDT responses.

In this exploratory study, a Go/NoGo decision framework was applied to support the structured assessment of observed PDT-induced biological effects and to estimate the probability of erroneous conclusions based on predefined response thresholds [47]. Therefore, three zones, i.e., a PDT Effect zone (Go), a No Effect zone (NoGo), and a so-called Consider zone, were pre-defined, as illustrated in Figure 7.

To avoid misinterpretation, we emphasize that the Go/NoGo classification used in this study does not represent therapeutic efficacy in a clinical or oncological sense. The predefined 50% viability threshold does not imply tumor eradication or clinical benefit. Instead, it serves as an internal, model-based metric for categorizing relative biological responses to PDT within the optical phantom model. The Go/NoGo zones therefore reflect the degree of PDT-induced inhibitory effects on spheroid viability rather than long-term survival or therapeutic success. Throughout the manuscript, these responses are interpreted as biological or PDT-induced inhibitory effects and not as clinical therapeutic effects.

Based on normalized cell viability as an inverse surrogate for PDT-induced biological response, a viability of less than 50% indicated a strong treatment effect and was categorized as a clear Go, reflecting PDT response. Conversely, a viability above 70% indicated insufficient treatment response and was categorized as a clear NoGo. For results falling into the Consider zone, i.e., from 50 to 70% cell viability, the assessment of the PDT response was complemented by additional qualitative criteria, such as microscopic imaging and morphological observations, to support a more nuanced interpretation.

In a positive scenario (base case), a cell viability, of 20% with a standard deviation of 10% was assumed, indicating a strong PDT response. In contrast, in a negative scenario (worst case), a cell viability, of 90% (standard deviation 10%) was assumed, indicating no PDT effect. Based on these assumptions derived from previous studies and preliminary experiments conducted by our research group, and the predefined zone boundaries, the probabilities of correct or incorrect classifications were calculated using software R (version 4.3.2).

With three evaluable samples, the probability of a correct classification as Go in the base case, which assumes a cell viability, of 20%, is 98%. In the worst case, defined by an assumed cell viability, of 90%, the probability of a correct classification as NoGo is 96%. Accordingly, the probabilities of incorrect classification are very low (2% and 4%, respectively), even when multiple classifications are made.

All data collected in this study were analyzed using descriptive statistics and visualized accordingly (absolute and relative frequencies or mean values including standard deviations).

## 5. Conclusions

For the first time, we have shown the feasibility of a synthetic model that, within the framework of PDT, simulates attenuation of light during its passage through brain tissue. This work has direct implications for the standardized evaluation of PDT in vitro. Our data confirm that a short single PDT session delivered by a potential implant such as the Globus Lucidus [26] is sufficient to have anti-neoplastic activity against GBM cells within a few millimeters around such an implant, when placed in the resection cavity following surgical removal of the primary tumor. This reflects the typical clinical presentation of glioblastoma, where residual infiltrating tumor cells are found in the peritumoral zone adjacent to the resection margin. Further studies are warranted to examine how alterations of the irradiation regimens may affect the therapeutic penetration depth and potentially improve the therapeutic efficacy against GBM. Whether this approach can contribute to improved treatment of glioma patients will need to be assessed in future preclinical and clinical studies.

## Figures and Tables

**Table 1:** Irradiance measurements were conducted of light passing through phantom plates of the indicated thicknesses, mimicking the optical properties of both white and gray brain matter sourced from pigs. These measurements were performed at a wavelength of 630 nm with an initial irradiance set at 42.25 mW/cm^2^. Dashes indicate absent measurements.

Thickness of Phantom Material [mm]	Irradiance “White Matter” [mW/cm^2^]	Irradiance “Gray Matter” [mW/cm^2^]
1	2.85	6.14
2	1.29	3.78
3	0.70	2.57
4	0.38	-
5	0.21	1.29
6	0.13	-
7	-	0.48
9	-	0.25

**Table 2:** Overview of the glioblastoma (GBM) cell models used in this study, including their origin, diagnosis, patient information (age, sex, and tumor location), MGMT status, and specific characteristics.

Cell line	Diagnosis	Location	Age at Diagnosis	Sex	MGMT	Specification
U251MG	Glioblastoma WHO IV	Left parieto-occipital	47	M	N/A	Established commercially available cell line
T98G	Glioblastoma WHO IV	N/A	61	M	+,[45]	Established commercially available cell line
SC38	Glioblastoma WHO IV	Right temporal	75	M	−,[44,45]	Established as spheroids from patient material
SC40	Glioblastoma WHO IV	Left frontal	57	F	−,[44,45]	Established as spheroids from patient material
PC38	Glioblastoma WHO IV	Right temporal	75	M	−,[44,45]	Differentiated from SC38 under exposure to FBS
PC40	Glioblastoma WHO IV	Left frontal	57	F	−,[44,45]	Differentiated from SC40 under exposure to FBS

## Data Availability

The original contributions presented in this study are included in the article. Further inquiries can be directed to the corresponding author.

## Abbreviations

The following abbreviations are used in this manuscript:

5-ALA	5-Aminolevulinic acid
ATP	Adenosine triphosphate
GBM	Glioblastoma
LED	Light-emitting diode
OS	Overall survival
PDT	Photodynamic therapy
PFS	Progression-free survival
PpIX	Protoporphyrin IX
ROS	Reactive oxygen species
